# PD46 - Serum level of S 100 proteins in patients with asthma

**DOI:** 10.1186/2045-7022-4-S1-P46

**Published:** 2014-02-28

**Authors:** Theodora Gioka, Theocharis Konstantinidis, Christina Tsigalou, Eleni Hatziioannou, Georgia Kampouromiti, Dimitrios Cassimos

**Affiliations:** 1 Immunology Department of Microbiology Laboratory, University General Hospital of Alexandroupolis, Alexandroupolis, Greece; 2 Pediatric Department, University General Hospital of Alexandroupolis, Alexandroupolis, Greece

## Aim

Asthma is a chronic disease of the lower respiratory tract that is characterized by inflammation and bronchial obstruction. The pathophysiology of the disease is related to constriction of the bronchial smooth muscle, something that is influenced by calcium homeostasis. Important role in calcium homeostasis, play various binding proteins, which belongs to the family of proteins S100 (S100). The purpose of this study was to measure S100 protein in a group of children with bronchial asthma (BA) in comparison with age-sex matched control group.

## Materials and methods

10 patients (mean age 8±4.6, 6♂ and 4♀) and 10 age-sex matched controls (mean age 7.4±3.1, 4♂ and 6♀) were included in our study; Patients with acute infection were excluded from this study in order to avoid blases that could lead to misinterpretation. Serum S100 protein was determined by electrochemeluminescent method and level of protein ≥ 0,105µg/l was considered positive.

## Results

All asthmatic patients (100%) had abnormal serum levels of S100. Asthmatic patients presented a significantly higher level of S100 in comparison to control subjects (0.31μg/l vs 0.065μg/l, p<0.05).

## Conclusions

Our results in this study group demonstrated that S 100 elevated in asthmatic children, in a statistical significant manner. The role of S100 needs to be further investigated, imposing a possible new therapeutic target in the pathophysiology of asthma.

